# Highly Effective Synthetic Polymer-Based Blockers of Non-Specific Interactions in Immunochemical Analyses

**DOI:** 10.3390/polym16060758

**Published:** 2024-03-10

**Authors:** Vladimír Šubr, Libor Kostka, Jan Plicka, Ondřej Sedláček, Tomáš Etrych

**Affiliations:** 1 Institute of Macromolecular Chemistry, Academy of Sciences, Heyrovského nám 2, 160 00 Prague, Czech Republic; subr@imc.cas.cz (V.Š.); kostka@imc.cas.cz (L.K.); 2 ELISA Development Ltd., Velké Žernoseky 186, 412 01 Litoměřice, Czech Republic; info@elisadevelopment.cz; 3 Sophomer Ltd., Radiová 1285/7, Hostivař, 102 00 Prague, Czech Republic; 4 Department of Physical and Macromolecular Chemistry, Faculty of Science, Charles University, 128 40 Prague, Czech Republic; sedlacek@natur.cuni.cz

**Keywords:** HPMA copolymer, blocker, non-specific interactions, immunochemical analyses, bovine serum albumin, TSH ELISA kit

## Abstract

In vitro diagnostic methods face non-specific interactions increasing their background level and influencing the efficacy and reproducibility. Currently, the most important and employed blocker of non-specific interactions is bovine serum albumin (BSA), an animal product with some disadvantages like its batch-to-batch variability and contamination with RNases. Herein, we developed amphiphilic water-soluble synthetic copolymers based on the highly biocompatible, non-immunogenic and nontoxic *N*-2-(hydroxypropyl)methacrylamide (HPMA)-based copolymers or poly(oxazoline)s as highly effective synthetic blockers of non-specific interactions and an effective BSA alternative. The highest blocking capacity was observed for HPMA-based polymers containing two hydrophobic anchors taking advantage of the combination of two structurally different hydrophobic molecules. Polymers prepared by free radical polymerisation with broader dispersity were slightly better in terms of surface covering. The sandwich ELISA evaluating human thyroid-stimulating Hormone in patient samples revealed that the designed polymers can fully replace BSA without compromising the assay results. Importantly, as a fully synthetic material, the developed polymers are fully animal pathogen-free; thus, they are highly important materials for further development.

## 1. Introduction

One of the critical complications of any immunochemical analysis is the non-specific binding of the reactants (analyte, sample components, antibody and the analyte-antibody complex) to the solid-phase surface. The non-specific binding (NSB) may reduce the assay sensitivity, specificity and reproducibility. Reactants that non-specifically bind to the solid phase are most often used to minimise NSB, for example, animal proteins such as bovine serum albumin (BSA), casein, skimmed milk, fish or pig gelatin [1,2,3]. However, a common disadvantage of these blockers is the considerable variability of individual production batches requiring costly testing of each batch by the end-users. Another disadvantage is the requirements for tests confirming the harmlessness of the animal pathogens or contaminants listed in the relevant legislation.

BSA, so-called Albumin Fraction V, is the most used NSB for immunochemical tests isolated in a way that destroys the enzymatic activities of proteases. BSA-biotin conjugates are also used to bind avidin or streptavidin and subsequently other biotinylated components such as antibodies or antigens. However, BSA is not an ideal molecule to suppress NSB, so alternative synthetic molecules such as detergents, i.e., Tween 20, or polymers, i.e., poly(vinyl alcohol) or Ficoll, have been used [1,4,5,6]. However, they do not sufficiently prevent NSB [7]; therefore, there is a huge demand for the development of more effective blocking systems.

Cationic surfactants based on structurally different poly(ethylene glycols) conjugated to alkylamines act as functional blocking agents [8,9]. In addition to modified PEGs, the use of modified poly(vinyl alcohol) reduces the amount of NSB of antibodies and poly(vinylpyrrolidone) increases the sensitivity of antibody detection [10]. Herein, we describe novel hydrophilic polymer-based synthetic macromolecular blockers of NSB capable of suppressing the non-specific sorption of antibodies or other macromolecules to the solid phase. The developed poly[*N*-2-(hydroxypropyl)methacrylamide] (HPMA)-based or poly(2-oxazoline)-based amphiphilic copolymers are highly biocompatible, non-immunogenic, nontoxic, soluble in aqueous solutions, animal pathogen-free, and enable the attachment of active molecules of different origins—thus are an alternative to BSA in diagnostic assays.

## 2. Materials and Methods

### 2.1. Materials

1-amino-propan-2-ol, methacryloyl anhydride, methacryloyl chloride, thiazolidin-2-thione, β-alanine, *N*-ethyl-N′-(3-dimethylaminopropyl)carbodiimide hydrochloride (EDC.HCl), carbon disulfide, ethanethiol, sodium hydride (60% dispersion in mineral oil), *N*,*N*-diisopropylethylamine (DIPEA), 4-(dimethylamino)pyridine (DMAP), tert-butanol (tert-BuOH), *N*,*N*-dimethylacetamide (DMAc), *N*,*N*-dimethylformamide (DMF), dichloromethane (DCM), dimethyl sulfoxide (DMSO), lithium hydroxide, *N*,*N*’-diisopropylcarbodiimide, 2-thiazoline-2-thiol, 2,2′-azobisisobutyronitrile (AIBN), S-(2-cyanoprop-2-yl)-S-dodecyltrithiocarbonate (dodecyl-trithio-AIBN), *N*-biotinyl-ethylenediamine trifluoroacetate salt (NH_2_-ED-biotin.CF_3_COOH) and HABA-Avidin Reagent kit were obtained from Merck (Prague, Czech Republic). Initiator 2,2′-azobis(4-methoxy-2,4-dimethylvaleronitrile) (V-70) was obtained from FUJIFILM Wako Chemicals Europe GmbH (Neuss, Germany). Dodecylamine, aminocyclooctane, 5-norbornene-2-methylamine and 3-aminoquinuclidine.2HCl were purchased from TCI EUROPE N.V. (Haven, Belgium). Methyl ester side chain-containing poly(2-methyl-2-oxazoline) P(MeOx_95_-stat-MestOx_5_)-N_3_, was synthesised according to reference [11]. 3,3’,5,5’-Tetramethylbenzidine (TMB) was purchased from Surmodics (Eden Prairie, MN, USA). BSA Protease Free was purchased from Technopole (Limoges, France). NHS-biotin was obtained from Thermo Scientific (Waltham, MA, USA). All chemicals and solvents were of analytical grade; all solvents were purchased as dried.

### 2.2. Synthesis of Monomers, Chain Transfer Agent and Polymer Precursors

#### 2.2.1. Synthesis of HPMA

The monomer HPMA was prepared by the reaction of 1-amino-2-propanol with methacryloyl anhydride in diethyl ether [12]. Briefly, 17.0 g of 1-amino-2-propanol (AP) was dissolved in 550 mL of dry diethyl ether before the addition of 0.1 g of the inhibitor 2,5-di-tert-butylhydroquinone. Then, 35 g of methacryloyl anhydride was dissolved in 150 mL of dry diethyl ether, and 0.1 g of inhibitor was added. The methacryloyl anhydride solution was added dropwise to the AP solution at room temperature so that the temperature did not exceed 25 °C to form a homogeneous solution which was stirred for 1 h at ambient temperature. The solution was cooled to −20 °C with constant stirring, and the product was filtered, washed twice with dried and cooled diethyl ether. Finally, the monomer was recrystallised from acetone (32.5 g/70 mL acetone) yielding 25.2 g (77%).

#### 2.2.2. Synthesis of 3-(3-Methacrylamido-propanoyl)thiazolidine-2-thione

Monomer 3-(3-methacrylamido-propanoyl)thiazolidine-2-thione (Ma-β-Ala-TT) was prepared according to the published procedure [13].

#### 2.2.3. Synthesis of Chain Transfer Agent N-[2-[5-[(3aR,4R,6aS)-2-oxo-1,3,3a,4,6,6a-hexahydrothieno [3,4-d]imidazol-4-yl]pentanoylamino]ethyl]-4-cyano-4-dodecylsulfanylcarbothioylsulfanyl-pentanamide (Dodecyl-trithiocarbonate-ED-biotin)

Dodecyl-trithiocarbonate-ED-biotin was prepared by a two-step synthesis. In the first step, 2-dodecylsulfanylcarbothioylsulfanyl-2-methyl-5-oxo-5-(2-thioxothiazolidin-3-yl)pentanenitrile (dodecyl-trithiocarbonate-TT) was prepared by reacting 4-cyano-4-dodecylsulfanylcarbothioylsulfanyl-pentanoic acid (dodecyl-trithiocarbonate-COOH) with thiazolidine-2-thione (TT) in DCM in the presence of EDC.HCl. In the second step, the prepared dodecyl-trithiocarbonate-TT was reacted with *N*-biotinyl-ethylenediamine trifluoroacetate in DMF to form the desired dodecyl-trithiocarbonate-ED-biotin.

Dodecyl-trithiocarbonate-COOH (100 mg, 0.247 mmol) and thiazolidine-2-thione (31.0 mg, 0.260 mmol) were dissolved in 4 mL of DCM, and a catalytic amount of 4-(dimethylamino)pyridine and EDC.HCl (57 mg, 0.297 mmol) was added to the solution. The reaction mixture was stirred for 4 h at room temperature.

**Figure 1:**
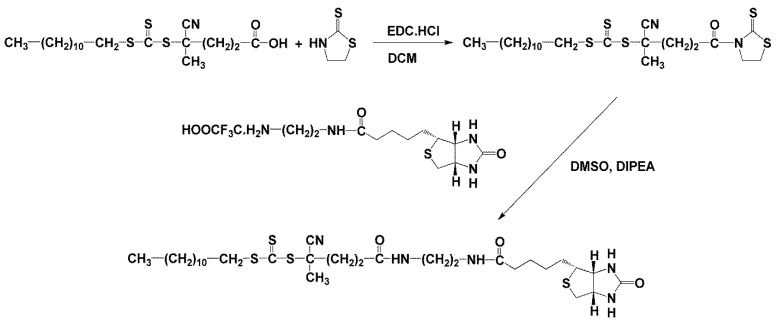
Synthesis of chain transfer agent dodecyl-trithiocarbonate-ED-biotin.

Dodecyl-trithiocarbonate-TT (100 mg, 0.247 mmol) was dissolved in 1 mL of DMF. NH_2_-ED-biotin.CF_3_COOH (99.15 mg, 0.247 mmol) was dissolved in 1 mL of DMF, and DIPEA (43 µL, 0.247 mmol) was added to the solution. The NH_2_-ED-biotin solution was then added to the dodecyl-trithiocarbonate-TT solution and stirred for 1 h at room temperature. During the reaction, the initially yellowish solution turned colourless. The reaction mixture was poured into 100 mL of distilled water, and the precipitate was isolated by centrifugation. The product was dissolved in isopropanol and evaporated in a vacuum to obtain solid dodecyl-trithiocarbonate-ED-biotin (see Figure 1).

The chain transfer agent S-2-cyano-2-propyl S’-ethyl trithiocarbonate (CTA-AIBN) was prepared according to the published procedure [14].

#### 2.2.4. Synthesis of Copolymer Precursors by RAFT Copolymerisation

Copolymer precursors pHPMA-co-Ma-β-Ala-TT (P1–P5) were prepared by reversible addition-fragmentation chain-transfer copolymerisation (RAFT) of HPMA and Ma-β-Ala-TT in the presence of chain transfer agent (CTA-AIBN) and initiator 2,2’-azobis(4-methoxy-2,4-dimethylvaleronitrile (V-70). The different molar mass of polymer precursor P1–P5 was controlled by changing the ratio of monomer:CTA from 100:1 to 650:1.

Copolymer precursor P3 was prepared by dissolving HPMA (1.25 g, 8.73 mmol) in 10.9 mL of tert-BuOH and mixing with Ma-β-Ala-TT (0.308 g, 1.19 mmol) dissolved in 2.5 mL of DMAc. Then, CTA-AIBN (3.13 mg, 1.53 × 10^−2^ mmol) and V-70 (7.63 × 10^−3^ mmol) were added, and the ratio of monomer/CTA/V-70 was 500/1/0.5. The polymerisation mixture was bubbled with argon for 10 min before the ampule was sealed and copolymerisation was performed at 40 °C for 24 h. The copolymer was isolated by precipitation into a mixture of acetone/diethyl ether 3/1, filtered and dried in a vacuum. The copolymer was dissolved in methanol and precipitated into acetone/diethyl ether 3/1, filtered and dried in a vacuum. The trithiocarbonate terminating groups were removed by the method described by Perrier [15]. The yield was 0.92 g of copolymer P3.

#### 2.2.5. Synthesis of Copolymer Precursor by Radical Solution Copolymerisation

Copolymer precursors pHPMA-co-Ma-β-Ala-TT (P6) were prepared by radical solution copolymerisation. Copolymer precursor P6 was synthesised as follows: HPMA (1.0 g, 6.98 mmol) and Ma-β-Ala-TT (0.246 g, 0.95 mmol) were dissolved in 7.8 mL of DMSO before the addition of the initiator (AIBN; 100 mg, 1 wt%). The polymerisation mixture was bubbled with argon for 10 min and then sealed in an ampule for copolymerisation at 60 °C for 6 h. Copolymer P6 was isolated by precipitation into a mixture of acetone/diethyl ether 3/1, filtered and dried in a vacuum. The copolymer was dissolved in methanol and reprecipitated into acetone/diethyl ether 3/1, filtered and dried in a vacuum yielding 0.965 g (77.4%) of copolymer P6.

#### 2.2.6. Synthesis of Copolymer Precursor by Radical Precipitation Copolymerisation

Copolymer precursor pHPMA-co-Ma-β-Ala-TT (P7) was prepared by radical precipitation copolymerisation. Briefly, HPMA (1.0 g, 6.98 mmol) and Ma-β-Ala-TT (0.246 g, 0.95 mmol) were dissolved in 7.8 mL of acetone before the addition of the initiator (AIBN) (100 mg, 1 wt%). The polymerisation mixture was bubbled with argon for 10 min and then sealed in an ampule for copolymerisation at 60 °C for 24 h. The precipitated copolymer was filtered, dried in a vacuum and dissolved in methanol before reprecipitating into acetone/diethyl ether 3/1, filtered and dried in a vacuum to yield 0.965 g (77.4%) of copolymer P7.

#### 2.2.7. Synthesis of p(MeOx_95_-stat-(COOH)Ox_5_)-N_3_

A solution of lithium hydroxide (10 eq. relative to the methyl ester groups amount) in distilled water (5 mL) was added to a solution of methyl ester-containing polymer precursors (800 mg) in distilled water (10 mL) and stirred at room temperature for 3 h. Then, the mixture was acidified by concentrated hydrochloric acid to pH ~3, transferred to dialysis tubing (1 kDa MWCO) and dialysed against distilled water for two days. The polymers were recovered by freeze-drying.

#### 2.2.8. Synthesis of pMeOx TT (P8)

**Figure 2:**
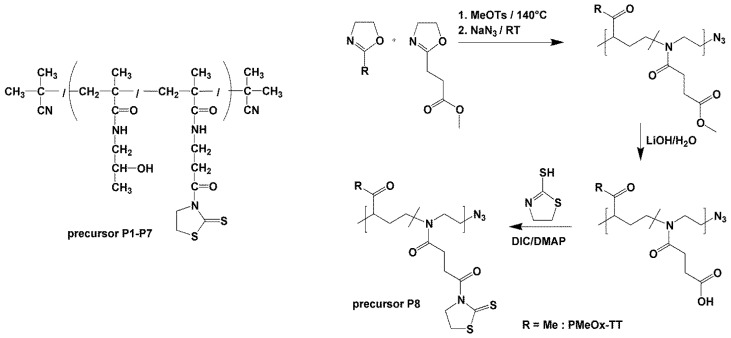
Structures of polymer precursors P1–P7 and synthesis of TT-containing poly(2-oxazoline) precursor P8.

The corresponding carboxylic acid-containing copolymers p(MeOx_95_-stat-(COOH)Ox_5_)-N_3_ (500 mg) were dissolved in *N*,*N*-dimethylformamide (10 mL) and cooled in an ice-water bath. A solution of *N*,*N*’-diisopropylcarbodiimide (2 eq. relative to the carboxylic acid groups amount) and 4-dimethylaminopyridine (0.1 eq.) in DMF (3 mL) was added dropwise, followed by the solution of 2-thiazoline-2-thione (2 eq.) in DMF (2 mL). The reaction mixture was then allowed to warm to room temperature and stirred overnight. The crude copolymers were isolated by precipitation into diethyl ether, filtered and dried in vacuo. Finally, the crude copolymer was purified by gel filtration using Sephadex LH-20 sorbent in methanol. The polymer-containing fractions were evaporated under a vacuum, dissolved in cold distilled water and freeze-dried. The copolymer was obtained as yellowish solids (yields 86% for PMeOx-TT). The TT group content (3.12 mol% for PMeOx-TT) was determined by ^1^H NMR spectroscopy, see Figure S1, in CDCl_3_ by comparing the integral areas of the TT peak at 4.6 ppm with the polymer backbone peak at 3.5 ppm. The polymer precursor structures and characteristics are shown in Figure 2 and Table 1.

### 2.3. Synthesis of Polymer Conjugates

#### 2.3.1. Synthesis of Heterotelechelic Polymer Conjugates C1–C3 Dodecyl-p(HPMA)-ED-biotin Using RAFT Polymerisation

Dodecyl-p(HPMA)-ED-biotin polymer conjugates were prepared by RAFT polymerisation. For example, conjugate C2 was synthesised by dissolving 0.4 g of HPMA in 3.4 mL of tert-BuOH and 18.8 mg of dodecyl-trithiocarbonate-ED-biotin in 425 µL of DMAc and mixing with 4.31 mg of initiator V-70 before transfer to a polymerisation ampoule. The mixture was bubbled with argon for 10 min and the ampoule was sealed for polymerisation at 30 °C for 72 h. The polymer conjugate was isolated by precipitation into acetone:diethyl ether (3:1), filtered, washed with acetone and diethyl ether and dried under a vacuum. The dodecyl-p(HPMA)-ED-biotin polymer conjugate had a molecular weight *M*_w_ = 22,400 g.mol^−1^, polydispersity *Ð* = 1.18 and contained one dodecyl molecule at the α-end of the pHPMA chain and one molecule of biotin at the ω-end of the polymer chain.

#### 2.3.2. Synthesis of Semitelechelic Dodecyl-p(HPMA) Conjugates C4–C6

**Figure 3:**
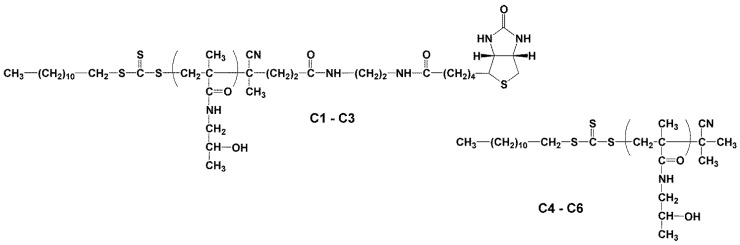
Structures of copolymer conjugates of dodecyl-p(HPMA)-biotin and dodecyl-p(HPMA).

The polymer conjugates dodecyl-p(HPMA) (C4, C5 and C6) were prepared by RAFT-polymerisation (reversible addition-fragmentation chain-transfer). For example, conjugate C5 was prepared by dissolving HPMA (0.4 g, mmol) in 3.4 mL of tert-BuOH and chain transfer agent of S-(2-cyanoprop-2-yl)-S-dodecyltrithiocarbonate (dodecyl-trithiocarbonate) (3.9 mg, 1.12 × 10^−2^ mmol) in 64 µL of DMAc before the addition of the initiator V-70 (1.72 mg, 5.59 × 10^−3^ mmol). The mixture was transferred to a polymerisation ampoule, bubbled with argon for 10 min and then sealed for polymerisation at 40 °C for 24 h. The polymer precursor was isolated by precipitation into acetone:diethyl ether (3:1), filtered, washed with acetone and diethyl ether and dried under a vacuum. The dodecylamine-p(HPMA) polymer conjugate had a molecular weight *M*_w_ = 30,000 g.mol^−1^, polydispersity *Ð* = 1.02 and contained one molecule of dodecyl at the α-end of the polymer chain. The polymer precursor structures and their characteristics are shown in Figure 3 and Table 2.

#### 2.3.3. Synthesis of Conjugates p(HPMA-co-Ma-β-Ala-dodecylamin-co-Ma-β-Ala-ED-biotin) (C7–C10)

Polymer conjugates C7–C9 were prepared by aminolytic reaction of polymer precursors p(HPMA-co-Ma-β-Ala-TT) (P1, P3 and P4) containing thiazolidine-2-thione reactive groups along the copolymer chain with dodecylamine and NH_2_-ED-biotin.CF_3_COOH in DMSO in the presence of DIPEA.

An example of polymer conjugate C8 synthesis: polymer precursor P3 p(HPMA-co-Ma-β-Ala-TT) (50 mg, 3.71 × 10^−2^ mmol TT), dodecylamine (0.8 mg, 4.32 × 10^−3^ mmol) and NH_2_-ED-biotin.CF_3_COOH (2.4 mg, 5.99 × 10^−3^ mmol) was dissolved in 0.25 mL DMSO. DIPEA (9.0 µL, 1.03 × 10^−2^ mmol) was then added and the reaction mixture was stirred for 4 h at room temperature. Subsequently, 1-amino-propan-2-ol (10 µL) was added to the solution and the reaction mixture was stirred for 10 min. Then the polymer conjugate C8 p(HPMA-co-Ma-β-Ala-dodecylamine-co-Ma-β-Ala-ED-biotin) was isolated by precipitation into a mixture of acetone/diethyl ether (3/1), filtered, washed with acetone and diethyl ether and dried in a vacuum to yield 38 mg of polymer.

#### 2.3.4. Synthesis of Polymer Conjugates p(HPMA-co-Ma-β-Ala-dodecylamin) (C11–C14)

Polymer conjugates C11–C14 were prepared by aminolytic reaction of polymer precursor p(HPMA-co-Ma-β-Ala-TT) (P3, P4 and P6) with dodecylamine in DMSO in the presence of DIPEA.

**Figure 4:**
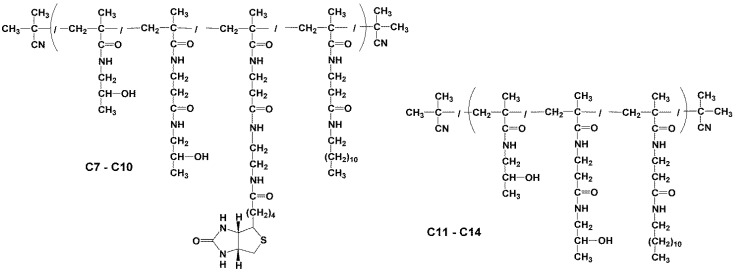
Structures of p(HPMA) copolymer conjugates C7–C14.

Polymer conjugate C11 was synthesised as follows: polymer precursor P3 p(HPMA-co-Ma-β-Ala-TT) (82 mg, 6.07 × 10^−2^ mmol TT) and dodecyl-amine (1.8 mg, 9.71 × 10^−3^ mmol) were dissolved in 0.35 mL of DMSO. DIPEA (4.2 μL, 2.43 × 10^−2^ mmol) was then added, and the reaction mixture was stirred for 4 h at room temperature. Subsequently, 1-amino-propan-2-ol (10 μL) was added to the solution, and the reaction mixture was stirred for 10 min. Then, the polymer conjugate C11 p(HPMA-co-Ma-β-Ala-dodecylamine) was isolated by precipitation into acetone/diethyl ether (3/1), filtered, washed with acetone and diethyl ether, and dried under a vacuum. The structures of the p(HPMA) copolymer conjugates C7–C14 are shown in Figure 4.

#### 2.3.5. Synthesis of Polymer Conjugate C15

Polymer precursor P8 (0.2 g, *M*_w_ = 12,400 g.mol^−1^, *Ð* = 1.13, 3.12% mol TT) was dissolved in 1.0 mL of DMSO and 100 µL (1.0 mg) of a stock solution of dodecylamine in CHCl_3_ (5 mg/500 µL CHCl_3_) and 110 µL (1.1 mg) of a stock solution of *N*-biotinyl-ethylenediamine trifluoroacetate in DMSO (5 mg/500 µL DMSO) before the addition of DIPEA (10 μL). The reaction mixture was stirred for 4 h at room temperature, 1-amino-propan-2-ol (10 μL) was added and the reaction mixture was stirred for 10 min. Conjugate 15 was isolated by precipitation into acetone:diethyl ether (3:1), filtered, washed with acetone and diethyl ether and dried in a vacuum.

#### 2.3.6. Synthesis of Conjugate C16—Poly(2-methyl-oxazolin-co-N’-dodecyl-N-ethyl-butanediamide-co-N’-2-hydroxypropyl-N-ethyl-butanediamide)

**Figure 5:**
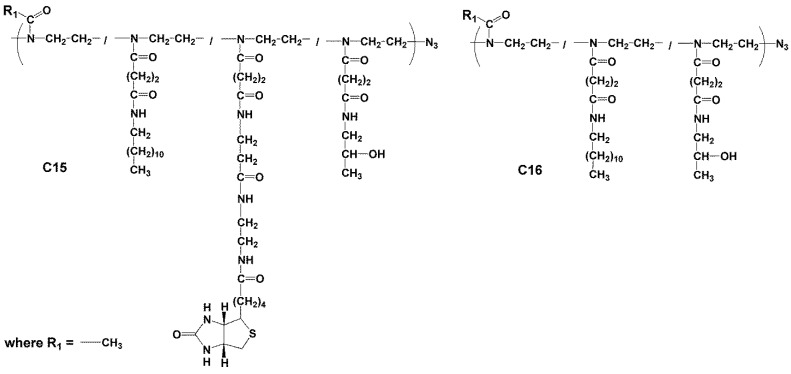
Structure of Pox conjugates C15–C16.

Polymer precursor P8 (0.2 g, *M*_w_ = 12,400 g.mol^−1^, *Ð* = 1.13, 3,12% mol TT) was dissolved in 1.0 mL of DMSO and 100 µL (1 mg) of a stock solution of dodecylamine in CHCl_3_ (1 mg/100 µL CHCl_3_) and DIPEA (5 μL) was then added, and the reaction mixture was stirred for 4 h at room temperature. Then, 1-amino-propan-2-ol (10 μL) was added to the solution, and the reaction mixture was stirred for 10 min. Conjugate 16 was isolated by precipitation into acetone:diethyl ether (3:1), filtered, washed with acetone and diethyl ether and dried in a vacuum. Structures of the poly(oxazoline) conjugates C15–C16 are shown in Figure 5. Characterisation of conjugates C7–C16 is summarised in Table 3.

#### 2.3.7. Synthesis of Polymer Conjugate C17—p(HPMA-co-Ma-β-Ala-dodecylamine-co-Ma-β-Ala-aminocyklooktane)

The polymer precursor P6 p(HPMA-co-Ma-β-Ala-TT) (200 mg, 1.63 × 10^−1^ mmol TT) was dissolved in 0.7 mL DMSO and 93 µL (4.4 mg, 2.37 × 10^−2^ mmol) of dodecylamine stock (23.66 mg/500 µL CHCl_3_) and 125 µL (2.18 mg, 1.71 × 10^−2^ mmol) of aminocyclooctane (CO) stock solution (8.7 mg/500 µL DMSO) was added. DIPEA (14.9 μL, 8.56 × 10^−2^ mmol) was added and the reaction mixture was stirred for 4 h at room temperature before the addition of 1-amino-propan-2-ol (10 μL) and the reaction mixture was stirred for 10 min. Then, the polymer conjugate C17 was isolated by precipitation into acetone:diethyl ether (3:1), filtered, washed with acetone and diethyl ether and dried in a vacuum.

#### 2.3.8. Synthesis of Polymer Conjugate C18—p(HPMA-co-Ma-β-Ala-dodecyl-amin-co-Ma-β-Ala-norbornen-2-methylamin)

The polymer precursor P6 p(HPMA-co-Ma-β-Ala-TT) (200 mg, 1.63 × 10^−1^ mmol TT) was dissolved in 1.0 mL DMSO and dodecylamine (4.4 mg, 2.37 × 10^−2^ mmol) and 5-norbornene-2-methylamine (NOR) (2.1 mg, 1.70 × 10^−2^ mmol) was added. DIPEA (14.8, 8.52 × 10^−2^ mmol) was added and the reaction mixture was stirred for 4 h at room temperature before the addition of 1-amino-propan-2-ol (10 μL) and the reaction mixture was stirred for 10 min. Then, the polymer conjugate C18 was isolated by precipitation into acetone:diethyl ether (3:1), filtered, washed with acetone and diethyl ether and dried under a vacuum.

#### 2.3.9. Synthesis of Polymer Conjugate C19—p(HPMA-co-Ma-β-Ala-dodecyl-amin-co-Ma-β-Ala-aminoquinuclidin)

Polymer precursor P7 p(HPMA-co-Ma-β-Ala-TT) (200 mg, 1.62 × 10^−1^ mmol TT) and dodecylamine (4.4 mg, 2.37 × 10^−2^ mmol) were dissolved in 1.0 mL DMSO. *N*,*N*-diisopropylethylamine (DIPEA) (10.3 μL, 5.93 × 10^−2^ mmol) was then added and the reaction mixture was stirred for 1 h at room temperature. Subsequently, 55 μL (1.71 × 10^−2^ mmol) stock solution of 3-aminoquinuclidine.2HCl (AQ) (12.41 mg/200 μL DMSO and DIPEA (14.9 μL, 8.54 × 10^−2^ mmol) were added to the solution and the reaction mixture was stirred for 24 h at room temperature. Then, 1-amino-propan-2-ol (10 μL) was added to the solution and the reaction mixture was stirred for 10 min. Polymer conjugate C19 was isolated by precipitation into acetone:diethyl ether (3:1), filtered, washed with acetone and diethyl ether and dried in a vacuum.

#### 2.3.10. Synthesis of Polymer Conjugate C20—p(HPMA-co-Ma-β-Ala-dodecyl-amin-co-Ma-β-Ala- 3-azabicyclo [3,3,0]octane)

**Figure 6:**
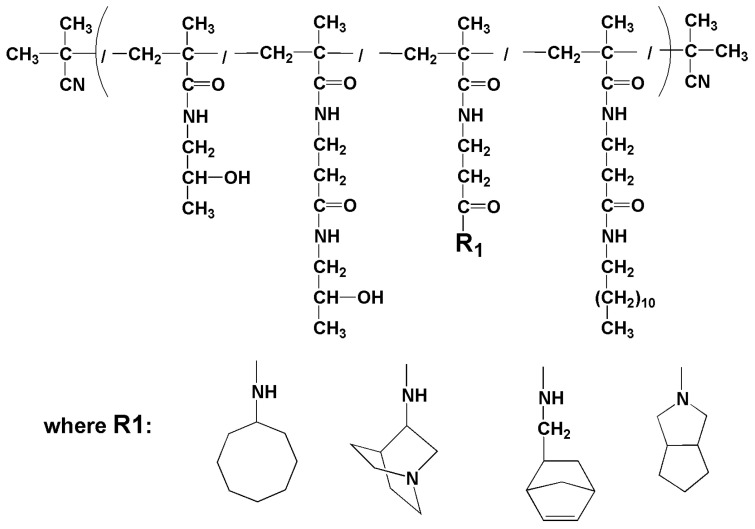
Structure of conjugates C17–C20.

Polymer precursor P7 p(HPMA-co-Ma-β-Ala-TT) (100 mg, 6.69 × 10^−2^ mmol TT) and dodecylamine (2.4 mg, 1.29 × 10^−2^ mmol) were dissolved in 0.4 mL DMSO. *N*,*N*-diisopropylethylamine (DIPEA) (5.1 μL, 2.46 × 10^−2^ mmol) was then added and the reaction mixture was stirred for 1 h at room temperature. Subsequently, 111.3 μL (2.37 × 10^−2^ mmol) stock solution of 3-Azabicyclo [3,3,0]octane.HCl (BA) (6.29 mg/200 DMSO) and DIPEA (14.9, 8.54 × 10^−2^ mmol) were added to the solution, and the reaction mixture was stirred for 24 h at room temperature. Then, 1-amino-propan-2-ol (10 μL) was added and the reaction mixture was stirred for 10 min before the polymer conjugate C20 was isolated by precipitation into acetone:diethyl ether (3:1), filtered, washed with acetone and diethyl ether and dried in a vacuum. The structures of conjugates C17–C20 are shown in Figure 6 and characterised in Table 4.

### 2.4. Characterisation of Polymer Precursors and Polymer Conjugates

Characterisation of monomers and chain transfer agents was conducted on an HPLC system (Shimadzu, Kyoto, Japan) equipped with a UV/Vis photodiode array detector and a reverse-phase column (Chromolith High-Resolution RP-18e, 100 × 4.6 mm) (Merck, Prague, Czech Republic). The mobile phase was 5–95% water–acetonitrile gradient with 0.1% TFA for 15 min at a flow rate of 1.0 mL/min. The molar mass of the monomers and CTAs was determined by mass spectrometry (MS LCQFleet, Thermo Fisher Scientific).

The content of TT reactive groups was determined by spectrophotometric analysis on a SPECORD 205 (Jena Analytics, Jena, Germany) at 305 nm (ε_305_ = 10 800 L·mol^−1^·cm^−1^; methanol).

Polymer precursors and conjugates were characterised by number-average molecular weight (*M*_n_), weight-average molecular weight (*M*_w_), and polydispersity (*Đ*) by size exclusion chromatography (SEC) on a system consisting of HPLC (Shimadzu, Kyoto, Japan) equipped with a UV detector and Wyatt Technology detectors: Optilab rEX differential refractometer and DAWN 8 multiangle light scattering detector. Column TSKgel G3000SW or TSKgel G4000SW with 20% 0.3 M acetate buffer (pH 6.5)/80% methanol (*v*/*v*) as a mobile phase was used for precursor characterisation.

Column Superose 6 with PBS (0.2 M, pH 7.4) as a mobile phase was used for conjugate characterisation.

The content of the dodecylamine, aminocyclooctane, 5-norbornene-2-methylamine and aminoquinuclidine in the polymer conjugates was determined in the hydrolysate (6 N HCl, 115 °C, 16 h) using HPLC with a fluorescence detector (Ex. 229 nm, Em. 490 nm) on a Chromolith C18 column by the method of pre-column derivatisation with o-naphthalenedialdehyde. The content of biotin-containing units was determined using the HABA-Avidin Reagent kit from Sigma-Aldrich.

The structures were confirmed by ^1^H NMR (300 MHz) using a Bruker DPX 300 spectrometer.

### 2.5. Evaluation in Diagnostic Methods

#### 2.5.1. Preparation of Antibody-Biotin Conjugate

The antibody-biotin conjugate was prepared by adding 1 mg of antibody to 1 mL borate buffer pH 8.5 biotinylated with 4% (*w*/*w*) Biotin-NHS (10 mg/mL in DMF) for 20 min. The reaction was terminated by the addition of 100 µL of 0.2 M NH_4_Cl solution and free biotin was separated by subsequent dialysis.

#### 2.5.2. Preparation of BSA-Biotin Conjugate

Briefly, 20 mg of BSA in 1 mL PBS buffer was biotinylated with 3% (*w*/*w*) Biotin-NHS added at a concentration of 20 mg.mL^−1^ in DMF for 60 min. The reaction was terminated by the addition of 100 µL of 0.2 M NH_4_Cl solution. Free biotin was separated by subsequent dialysis.

#### 2.5.3. Preparation of Horseradish-Peroxidase Conjugate

The antibody (1 mg) was conjugated to 5 mg of horseradish peroxidase (HRP) using glutaraldehyde. First, HRP was activated with 1.25% glutaraldehyde solution in phosphate buffer pH 6.8 for 14 h. After gel chromatography (Sephadex 25) to remove free glutaraldehyde, the activated HRP was mixed with a solution of IgG in 0.15 M NaCl with the addition of 5% (*v*/*v*) carbonate-bicarbonate buffer pH 9.5. The reaction was stopped after 14 h by the addition of a 5% (*v*/*v*) solution of 0.2 M lysine. The IgG-HRP conjugate was separated by gel chromatography on a Superdex HR 200 column. The conjugates were then used to construct two sandwich ELISA systems.

#### 2.5.4. Sorption of Biotinylated Conjugates

PBS with a conjugate containing biotin (conjugates C1, C2, C3, C7, C8, C9) and BSA as a competitor were aliquoted (200 µL) into a 96-well polystyrene microtiter plate (Greiner). The concentration of the conjugates C1, C2 and C3 was 1 mg/L, 0.1 mg/L for conjugates C7, C8 and C9, and 100 mg/L BSA. The plate was incubated at ambient temperature for 48 h without shaking. Subsequently, the solution was aspirated and 250 µL of PBS containing 0.1% fish gelatin was added to all wells and incubated overnight. After aspiration and repeated washing of the wells (3 × 350 µL) with a buffer containing Tween 20 and NaCl, 200 µL of commercial streptavidin conjugate with horseradish peroxidase (STR-HRP) diluted in phosphate buffer with 0.1% fish gelatin was added to each well and incubated for 1 h at room temperature without shaking. The wells were aspirated and washed (3 × 350 µL) before the addition of 200 µL of TMB. After 5 min, the colorimetric reaction was stopped by the addition of 50 µL of 2 M HCl. Subsequently, the absorbance was measured at a wavelength of 450 nm (Tables S1 and S2).

#### 2.5.5. Sorption of Non-Biotinylated Conjugates

The polyclonal rabbit antibody-HRP conjugate was diluted 2000 times in phosphate buffer containing 0.125 g/L BSA and 0.0125 g/L of conjugates C4, C5, C13 and C14. The test solutions were aliquoted (200 µL) into four wells of a 96-well polystyrene microtiter plate (Greiner). The plate was incubated for 60 min at room temperature without shaking. After aspirating and repeatedly washing the wells (3 × 350 µL) with buffer containing Tween 20 and NaCl, the colorimetric reaction was induced by adding 200 µL TMB. After 10 min, the colorimetric reaction was stopped by the addition of 50 µL of 2 M HCl. Subsequently, the absorbance (optical density OD) was measured at 450 nm (Table 5). The experiment comparing 1.0 g/L BSA and 0.1 g/L of conjugates C10, C14, C15, C16, C17, C18, C19 and C20 was performed in the same manner with 500 times dilution of the polyclonal rabbit antibody-HRP conjugate.

#### 2.5.6. ELISA of Human Thyroid Stimulating Hormone (hTSH) in Serum

A sandwich ELISA system for the determination of human hTSH was assembled from two commercially available mouse monoclonal antibodies. One monoclonal antibody was conjugated with biotin and the other with horseradish peroxidase.

#### 2.5.7. System with BSA

The biotin-labelled monoclonal antibody was bound to the wells of a microtiter plate using biotinylated BSA as follows: 300 µL of biotinylated BSA was pipetted into the wells of a microtiter plate (Greiner) and incubated overnight at room temperature. The solution was aspirated and 300 µL of a 1 µg/mL streptavidin solution in phosphate buffer was added and incubated overnight at room temperature. Then, the solution was aspirated and 250 µL of a biotinylated monoclonal antibody solution (1 µg/mL) in a phosphate buffer containing 1 mg/mL BSA was added and incubated for 48 h. The solution was removed by suction and the plate was air-dried for 4 h. The plates were now ready for use and were stored in the refrigerator for up to 6 months. The IgG-HRP conjugate was diluted 20,000× in citrate buffer containing BSA at a concentration of 1 mg/mL.

#### 2.5.8. System with Conjugates C8 and C14

The biotin-labelled monoclonal antibody was bound to the wells using conjugates C8 and C14 in a similar procedure to the BSA system with slight modifications: (i) C8 was used at a concentration of 0.1 µg/mL instead of 10 µg/mL biotinylated BSA; (ii) the biotinylated antibody coating solution contained 1 mg/mL of conjugate C8 instead of BSA. The IgG-HRP conjugate was diluted 20,000× in citrate buffer containing 1 mg/mL C14.

The same calibrators were used in both systems, i.e., hTSH (Sigma-Aldrich no. T9265) in bovine serum and the concentration was determined using the WHO reference preparer (NIBSC 80/558). The working protocol was also the same for both systems:

- 50 µL of calibrator or sample + 150 µL of IgG-HRP conjugate solution was pipetted into each well
- 120 min incubation at laboratory temperature without shaking
- the solution was aspirated and washed 3 times with washing solution
- 200 µL TMB was added and incubated for 10 min at room temperature in the dark
- 50 µL of 2 M HCl was added and the optical density was measured at 450 nm (OD450)

Table 6 shows the OD450 values of the calibrators for both systems, each calibrator was measured in duplicate.

## 3. Results and Discussions

Herein, we described the synthesis of fully synthetic copolymers based on pHPMA copolymers or poly(2-oxazoline) which can suppress non-specific interactions and thus be used as a potential replacement for BSA or other proteins in diagnostic assays.

**Figure 7:**
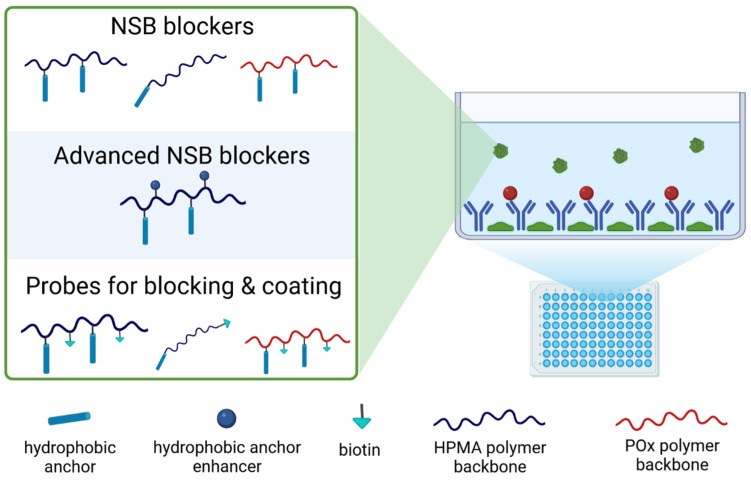
Schematic description of the developed polymer blockers.

A broad range of amphiphilic pHPMA or poly(2-oxazoline) conjugates containing dodecyl chains as functional anchors were synthesised and evaluated as NSB blockers. Moreover, advanced NSB blockers were enriched with a hydrophobic anchor enhancer to increase the blocking efficacy (Figure 6). A schematic overview of all developed synthetic NSB blockers is shown in Figure 7.

### 3.1. Synthesis of Polymer Precursors

Different types of radical polymerisation were applied to synthesise copolymer precursors pHPMA-co-Ma-b-Ala-TT (P1–P7, Figure 1). RAFT copolymerisation was employed for the synthesis of polymer precursors P1–P5 with low dispersity (≤1.2), differing in their molecular weights (11,000 to 74,000 g.mol^−1^). All polymer precursors contained a sufficient amount of reactive TT groups for subsequent reaction steps. Free radical polymerisation (FRP) was used to synthesise polymer precursors P6 and P7 either via solution polymerisation in DMSO (P6) or precipitation polymerisation in acetone (P7). FRP polymerisation yielded polymer precursors with a molecular weight of ~40,000 g.mol^−1^, relatively high dispersity (~2) and a sufficient amount of TT groups (Table 1). Polyoxazoline copolymer was employed for the synthesis of precursor containing the reactive TT groups. Cationic polymerisation was employed to synthesise poly(2-methyl-oxazoline) (P8) with a molecular weight of 20,000 g.mol^−1^. Subsequently, the TT groups were introduced by the replacement of methyl ester. The TT content in P8 was two to three times lower compared to the other HPMA-based copolymers but was sufficient for the subsequent conjugate synthesis. In summary, various polymer precursors differing in polymer type, molecular weight and dispersity were prepared for the synthesis of active polymer NSB blockers.

### 3.2. Synthesis of Polymer Conjugates

The polymer conjugates were synthesised by two different methods. The hydrophobic dodecyl anchors were introduced as follows: (i) in the side chain of the polymer precursor by aminolytic reaction of TT groups on the polymers with a dodecylamine amino group, (ii) to the main chain α-end of the polymer using functionalised CTA (monosubstituted NSB blockers). Importantly, the biotin was added in some cases in the side chains of the copolymers or to the main chain ω-end of the copolymer, thus forming probes for both blocking and coating.

### 3.3. NSB Blockers

The semitelechelic NSB conjugates containing dodecyl molecule at α-end chain C4–C6 (dodecyl-p(HPMA)) were synthesised by RAFT polymerisation using chain transfer agent dodecyl-trithiocarbonate with a molecular weight in the range 11,000–58,000 g.mol^−1^ and low dispersity. The schematic structures of p(HPMA) conjugates C4–C6 are depicted in Figure 3. Thus, for all polymer conjugates containing only one dodecyl molecule per polymer chain, the mass content of active molecules decreased several-fold with increasing molecular weight, see Table 2.

The conjugates C11–C14 with multiple dodecyl anchors along the polymer backbone were prepared from p(HPMA-co-Ma-β-Ala-TT) precursors P3–P6 and conjugate C16 was prepared from poly(oxazoline) precursor P8. These conjugates were synthesised by an aminolytic reaction of copolymer precursors containing thiazolidine-2-thione reactive groups along the polymer chain with dodecylamine. The conjugates C11–C14 based on the HPMA copolymers had a higher content of dodecyl molecules compared to semitelechelic conjugates C4–C6. The content of dodecyl molecules in conjugate C16 was around 0.94 wt% and was lower than in conjugates C11–C14. The schematic structures of p(HPMA) conjugates C4–C6 are depicted in Figure 3, conjugates C11–C14 in Figure 4 and conjugate C16 in Figure 5.

### 3.4. Probes for Blocking and Coating

The heterotelechelic NSB conjugates C1–C3 (dodecyl-p(HPMA)-ED-biotin) containing dodecyl and ED-biotin were synthesised by RAFT polymerisation using a new chain transfer agent dodecyl-trithiocarbonate-ED-biotin, see Figure 1. The conjugates C1–C3 containing at chain α-end dodecyl and chain ω-end ED-biotin were synthesised with a molecular weight in the range 18,000–58,000 g.mol^−1^ and low dispersity. All polymer conjugates C1–C3 contained one dodecyl and one ED-biotin molecule per polymer chain, thus the weight content of active molecules decreased several times with increasing molecular weight, see Table 2.

Moreover, conjugates C7–C10 and C15 consisted of dodecylamine and ED-biotin, which were introduced by the same type of aminolytic reaction as p(HPMA-co-Ma-β-Ala-TT) precursors (P1, P3, P4 and P6) and poly(oxazoline) precursor P8 with dodecylamine and NH2-ED-biotin. The conjugates C7–C10 based on the HPMA copolymers had a higher content of dodecyl molecules and biotins compared to conjugates C1–C3 and C15. The higher content of TT reactive side chain groups in p(HPMA-co-Ma-β-Ala-TT) precursors enabled higher loading of bioactive molecules than the sole functionalisation of the main chain ends (Table 3). Indeed, the bioactive molecules content was much lower for the poly(2-oxazoline) conjugates C15–C16, which was caused by the lower TT group content in precursor P8 (Table 1).

### 3.5. Advanced NSB Blockers

To increase the efficacy of the non-specific interaction blocking, conjugates C17–C20 containing dodecylamine and other hydrophobic molecules such as aminocyclooctane (CO), 5-norbornene-2-methylamine (NOR), 3-aminoquinuclidine (AQ) or 3-Azabicyclo [3,3,0]octane (AB) were synthesised from copolymer precursor P6 or P7 by the aminolytic reaction of TT reactive groups. The dodecylamine content in all four conjugates was around 1 wt%, the content of aminocyclooctane and 3-aminoquinuclidine in conjugates C17 and C19 was around 0.9 wt%, while the 5-norbornene-2-methylamine content in conjugate C18 was higher (see Table 4).

### 3.6. Competitive Assay with BSA for Biotinylated Conjugates

A competitive assay was performed to compare the blocking activity of the biotinylated polymers with one terminal hydrophobic anchor, polymers C1, C2 and C3, and polymers containing multiple anchors along the chain, polymers C7, C8 and C9.

**Figure 8:**
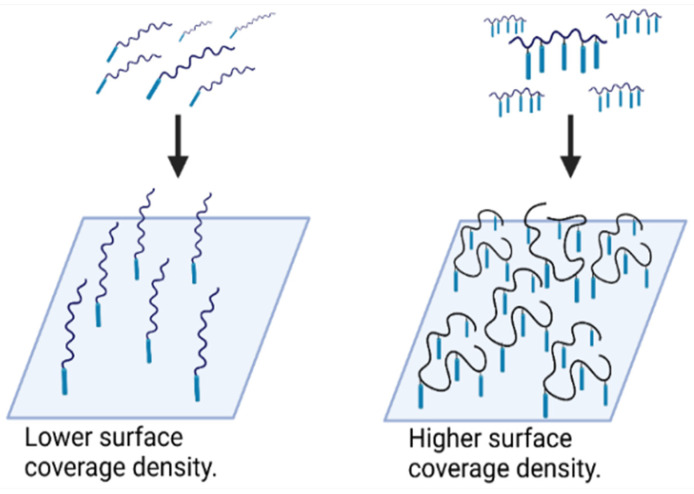
Schematic visualization of the PS surface covered by polymers with multiple anchor presentation and polymer with one anchor.

Based on preliminary experiments, the coating polymer concentration with one anchor was 10 times higher than the coating concentration of conjugates with multiple anchors, i.e., 1 mg/mL for polymers C1, C2 and C3 and 0.1 mg/mL for polymers C7, C8 and C9. The lower coating concentration of polymers with multiple anchors, 5 per chain, is due to their ability to cover the surface with the entire biocompatible chain (see Figure 8). There was no significant difference between the polymers with one terminal anchor, indicating that the molecular weight of these systems has no effect on their blocking function (Table S1). The blocking activity was halved with a 30-fold excess of BSA for all polymers. The size of the polymer chain plays a role in the case of polymers with multiple anchors (Table S2), with the shortest polymer C9 having lower coating activity than polymers C7 and C8 for which the coating activity was similar. Half the blocking activity was achieved at a 250-fold excess of BSA, thus showing the excellent coating efficacy of the developed polymers. In summary, polymers with multiple anchors are effective blocking agents compared to BSA, with the best coating polymers being C7 and C8 with a high molecular weight and multiple hydrophobic anchors. These biotinylated polymers are suitable for the coating of PS surfaces and the preparation of sandwich ELISAs.

### 3.7. The Ability of Non-Biotinylated Conjugates as NSB Reagents

The ability of non-biotinylated polymer conjugates to block the nonspecific sorption of polyclonal rabbit antibody conjugated to HRP to the PS surface was studied with four polymers, i.e., C4 and C5 polymers with one terminal anchor and C13 and C14 containing multiple anchors. As these NSB blockers are also intended for the blocking of non-specific interaction in solution, the number of anchors was reduced to two to prevent micelle formation which can negatively influence the blocking activity.

As the polyclonal rabbit antibody conjugated to HRP was diluted 2000 times, the polymer concentration was reduced to 0.0125 mg/L compared to the 10 times higher BSA concentration. Interestingly, the lower molecular weight of polymer C4 with one terminal hydrophobic anchor increased the blocking activity compared to polymers C13 and C14 containing multiple anchors. The mean optical densities of the three polymers were comparable to BSA (Table 5). The similar blocking activity of polymers C13 and C14 showed that the dispersity of the polymers prepared via controlled RAFT polymerisation (C13) or FRP (C14) do not play significant role in the blocking activity. In summary, polymers C4, C13 and C14 demonstrated good blocking activity for the PS surface even when used at a 10 times lower concentration than BSA.

### 3.8. Determination of hTSH in Human Serum Samples

A model ELISA kit was constructed for the determination of hTSH, a routine endocrine assay, in 500 anonymised human serum samples (obtained from Sophomer, s.r.o. and the Institute of Endocrinology in Prague, Czech Republic) to compare the properties of the developed polymers and BSA. Two variants of the TSH ELISA were designed, the first variant (V1) only used biotinylated and pure BSA and the V2 used the developed polymers (C8 as the biotinylated version and C14 as an NSB blocker). First, the TSH calibration showed that the OD450 values of both assays were comparable (Table 6) but the calibration responses were slightly higher for V2 showing the benefit of the polymer-based systems. It is important to note that to achieve the same calibration curve, it was necessary to use a 100-fold higher concentration of biotinylated BSA than polymer C8 and a 10 times higher concentration of BSA than polymer C14. The benefit of the V2 containing the polymers is visible in the lower mean OD450 value at 0 mIU/mL of TSH, with a greater difference between the values at 0 and 0.1 mIU/mL. The polymer-based V2 performed better in the TSH calibration than the classical BSA assay.

**Figure 9:**
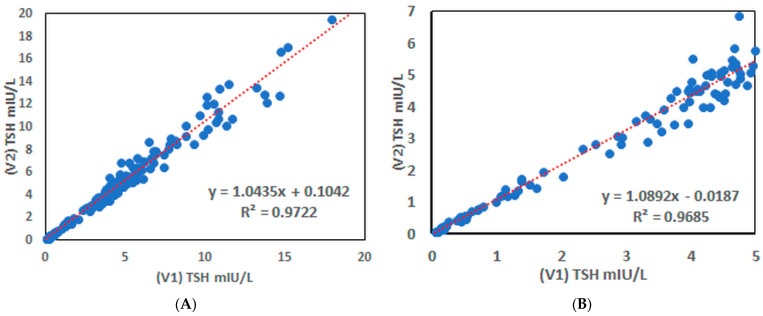
Correlation of the determined TSH values between the variant V1 employing the BSA and the variant V2 employing developed polymer blockers. (**A**)—full scale comparison; (**B**)—zoomed area from Figure 9A for the range of clinically normal TSH values.

The results of both variants for the 500 human serum samples are compared graphically in Figure 9A (entire TSH concentration range) and Figure 9B (clinically normal TSH range), showing that the TSH contents obtained by both variants were comparable. It is of note that conjugate C8 was used at a 100 times lower concentration than BSA and polymer C14 at a 10 times lower concentration than BSA. The linear correlation of the results of both variants is very good and meets the requirements for the clinical evaluation using both settings. We can conclude that the combination of the polymer blockers enables to build the assay setting with significantly lower amount of used materials with the same sensitivity and accuracy.

### 3.9. Advanced Polymer Blocker Evaluation

To increase the efficacy of the developed polymers, advanced polymer blockers were synthesised using either poly(oxazoline) chains, i.e., polymers C15 and C16, HPMA-based systems enriched with secondary hydrophobic molecules, i.e., aminocyclooctane for C17, 5-norbornene-2-methylamine for C18, 3-aminoquinuclidine for C19 or 3-azabicyclo [3,3,0]octane for C20, or different type of polymerisation, i.e., C10 and C14 prepared by FRP and C8, C11 and C12 prepared by RAFT.

Here, the polyclonal rabbit antibody-HRP conjugate was diluted 500 times so the polymer concentration was adjusted to 0.1 mg/L and the BSA concentration was 10 times higher. There was high variability in the inhibition of IgG-HRP conjugate sorption when using BSA as a blocker. All developed polymer conjugates demonstrated better blocking activity than BSA, with similar results achieved at a 10 times lower concentration. Of the conjugates tested, conjugates C20 and C17 were most effective, followed by conjugates C10, C12, C14, C18 and C19. Conjugates C8, C11, C15 and C16 were the least effective blockers. Importantly, the application of FRP for the synthesis of biotinylated polymer increased the blocking efficacy (compare C8 and C10 in Table 7). These results indicate that the polymer chain size can affect the blocking efficacy, with larger polymer chains better coating the entire PS surface, whereas the increased number of hydrophobic anchors in C11 decreased the blocking efficacy. The formation of stable micellar structures can play a significant role, so fewer hydrophobic anchors are preferable for blocking NSB. The application of poly(methyl oxazolin)s did not improve the blocking efficacy, with polymers C15 and C16 being less effective compared to the HPMA-based systems. Indeed, the combination of two hydrophobic anchors with different structures, polymers C17 and C20, demonstrated the best blocking efficacy.

Taken together, these results suggest that the developed polymers are much better blockers of NSB than BSA. The optimal polymer structure should contain two hydrophobic anchors that increase the blocking efficacy of the PS surface. Polymers prepared by FRP with broader dispersity may provide better surface cover.

## 4. Conclusions

Amphiphilic water-soluble synthetic copolymers based on the highly biocompatible, non-immunogenic, non-toxic HPMA-based copolymers or POXs were developed and confirmed as highly effective blockers of non-specific interactions. The developed polymers showed similar blocking activity to BSA but at much lower concentrations and, thus are more effective NSB blockers than most commonly employed blockers. The optimal polymer structure contains two different hydrophobic anchors that increase the blocking efficacy of the PS surface. Polymers prepared by FRP with broader dispersity provided better surface covering than the polymer prepared by controlled RAFT polymerization. The sandwich ELISA system evaluating hTSH in human serum samples demonstrated that the developed polymers can fully replace BSA and can be used at a significantly lower concentration. Importantly, the developed polymers are fully animal pathogen-free, thus they are highly important materials for further development.

## Figures and Tables

**Table 1:** Characterisation of copolymer precursors.

Precursor	Polymerisation	*M*_w_ [g.mol^−1^]	*M*_n_ [g.mol^−1^]	*Ð*	TT Content mol%
P1	RAFT	74,000	58,000	1.25	13.8
P2	RAFT	52,000	51,000	1.02	9.6
P3	RAFT	49,000	40,000	1.23	11.6
P4	RAFT	26,000	25,000	1.04	10.3
P5	RAFT	11,000	10,000	1.09	8.1
P6	Solution FRP	38,000	18,000	2.05	12.8
P7	Precipitation FRP	41,000	20,000	2.08	11.8
P8	P-MeOx	12,400	10,100	1.13	3.12

**Table 2:** Characterisation of heterotelechelic and semitelechelic p(HPMA) conjugates.

Conjugates	*M*_w_ g.mol^−1^	*M*_n_ g.mol^−1^	*Ð*	Dodecyl Content wt%	Biotin Content wt%
C1	58,000	52,000	1.12	0.36	0.29
C2	22,000	19,000	1.18	0.97	0.67
C3	18,000	17,500	1.01	1.05	0.74
C4	11,000	11,000	1.02	1.7	-
C5	30,000	29,000	1.02	0.64	-
C6	58,000	57,000	1.02	0.32	-

**Table 3:** Characterisation of p(HPMA) and poly(2-oxazoline) conjugates with dodecylamine and biotin along the polymer chain.

Conjugates	Precursor	*M*_w_ g.mol^−1^	*M*_n_ g.mol^−1^	*Ð*	DDA wt%	DDA per Chain	Biotin wt%
C7	P1	95,000	77,000	1.23	1.19	5.0	2.24
C8	P3	59,000	57,000	1.04	1.61	5.0	2.26
C9	P4	42,000	34,000	1.24	2.77	5.0	2.91
C10	P6	52,000	36,000	1.46	2.84	5.5	1.94
C11	P3	66,000	61,000	1.08	2.77	9.0	-
C12	P4	43,000	35,000	1.23	1.55	2.0	-
C13	P5	16,000	15,000	1.08	2.50	2.0	-
C14	P6	34,000	20,000	1.67	1.83	2.0	-
C15 ^a^	P8	14,000	12,300	1.14	1.24	0.8	1.03
C16 ^a^	P8	14,000	12,300	1.14	0.94	0.6	-

^a^ poly(2-oxazoline) based conjugates.

**Table 4:** Characterisation of p(HPMA) copolymer conjugates C17–C20.

Conjugates	Precursor	*M*_w_ g.mol^−1^	*M*_n_ g.mol^−1^	*Ð*	DDA wt%	CO wt%	NOR wt%	AQ wt%	BA wt%
C17 (F3-CO)	P6	43,000	30,000	1.43	1.03	0.85	-	-	
C18 (F3-NOR)	P6	43,000	30,000	1.43	0.96	-	2.3	-	
C19 (F3-AQ)	P7	39,000	27,000	1.44	1.05	-	-	0.87	
C20 (F3-BA)	P7	52,000	28,000	1.74	1.27	-	-	-	3.3 ^+^

^+^ Theoretical content from feed ratio.

**Table 5:** The ability of non-biotinylated conjugates to serve as NSB reagents.

	Conjugate C4	Conjugate C5	Conjugate C13	Conjugate C14	BSA
	**0.0125 g/L**				**0.125 g/L**
	**OD_450_**				
	1.357	2.471	1.748	1.821	1.304
	1.437	2.416	1.624	1.747	1.288
	1.446	2.485	1.587	1.872	1.227
	1.403	2.485	1.656	1.843	1.227
Mean	1.411	2.464	1.654	1.821	1.261
C.V., % *	2.86	1.34	4.17	2.93	3.22

* C.V. = Coefficient of Variability, was calculated as the standard deviation of a set of measurements divided by the mean of the set, times 100.

**Table 6:** TSH calibration dependence using blockers based on commercial BSA (biotinylated BSA in concentration 10 µg/mL and BSA in concentration 1 mg/mL) and the combination of conjugates C8 and C14 (0.1 µg/mL for C8 and 0.1 mg/mL for C14).

Calibrator TSH, mIU/L	System with BSA (V1)		System with Conjugates C8 and C14 (V2)	
	Mean OD_450_	C.V. *, %	Mean OD_450_	C.V. *, %
0	0.043	2.64	0.041	0.17
0.1	0.048	1.18	0.051	1.96
0.5	0.082	0.52	0.093	1.06
2.5	0.269	2.50	0.330	3.92
10	0.899	3.30	1.123	1.53
50	3.416	1.51	3.827	1.83

* C.V. = Coefficient of Variability, was calculated as the standard deviation of a set of measurements divided by the mean of the set, times 100.

**Table 7:** Inhibition of non-specific binding of polyclonal rabbit antibody labelled with horseradish peroxidase to the PS surface.

	BSA 1.0 g/L	Conjugate										
		C8	C10	C11	C12	C14	C15	C16	C17	C18	C19	C20
		0.1 g/L										
	OD_450_											
	0.699	0.992	0.732	0.850	0.530	0.687	0.987	1.090	0.592	0.805	0.895	0.521
	0.555	0.967	0.626	0.881	0.527	0.664	1.118	1.156	0.652	0.800	0.851	0.540
	0.879	1.051	0.625	1.026	0.634	0.698	1.138	1.062	0.565	0.789	0.811	0.527
	1.012	1.034	0.697	0.938	0.618	0.699	1.025	1.048	0.592	0.665	0.738	0.536
	0.995	1.003	0.639	0.920	0.539	0.682	1.016	1.066	0.688	0.640	0.712	0.512
	1.004	1.003	0.681	0.970	0.572	0.720	1.140	1.091	0.550	0.717	0.770	0.527
	0.737	0.990	0.619	0.963	0.570	0.651	1.143	1.090	0.611	0.665	0.698	0.517
	0.862	0.984	0.666	0.967	0.584	0.680	1.093	1.036	0.543	0.626	0.732	0.519
Mean	0.843	1.003	0.661	0.939	0.599	0.685	1.083	1.080	0.570	0.714	0.776	0.525
C.V. *, %	19.77	2.73	6.14	5.90	6.95	3.12	5.87	3.43	8.40	10.51	9.05	1.79

* C.V. = Coefficient of Variability, was calculated as the standard deviation of a set of measurements divided by the mean of the set, times 100.

## Data Availability

Data are contained within the article.

## Supplementary Materials

The following supporting information can be downloaded at: https://www.mdpi.com/article/10.3390/polym16060758/s1, Figure S1: 1H NMR spectra of PMeOx-TT (P8) polymeric precursors. Measured in CDCl3; Table S1: Results of competitive assay. First, polymers with one terminal hydrophobic anchor, polymers C1, C2 and C3, were coated to the PS surface and BSA with increasing concentration was used to detach the polymer coating; Table S2: Results of competitive assay. First, polymers with one multiple presentation of hydrophobic anchor, polymers C7, C8 and C9, were coated to the PS surface and BSA with increasing concentration was used to detach the polymer coating.
