# Supplementary material for: Highly Effective Synthetic Polymer-Based Blockers of Non-Specific Interactions in Immunochemical Analyses
Source: Polymers (Basel). 2024 Mar 10;16(6):758. doi: 10.3390/polym16060758 (PMC10974723; doi:10.3390/polym16060758)
Supplement: Supplementary file 1 [file polymers-16-00758-s001.zip › polymers-2887055-supplementary.pdf]

Supporting Information

Highly Effective Synthetic Polymer-Based Blockers of Non-Specific Interactions in Immunochemical Analyses

Vladimír Šubr [1], Libor Kostka [1], Jan Plicka [2,3], Ondřej Sedláček [4] and Tomáš Etrych [1,*]

[1] Institute of Macromolecular Chemistry, Academy of Sciences, Heyrovského nám 2, 160 00 Prague, Czech Republic
[2] ELISA Development Ltd., Velké Žernoseky 186, 412 01 Litoměřice, Czech Republic
[3] Sophomer Ltd., Radiová 1285/7, Hostivař, 102 00 Prague, Czech Republic
[4] Department of Physical and Macromolecular Chemistry, Faculty of Science, Charles University, 128 40 Prague, Czech Republic
* Correspondence: etrych@imc.cas.cz

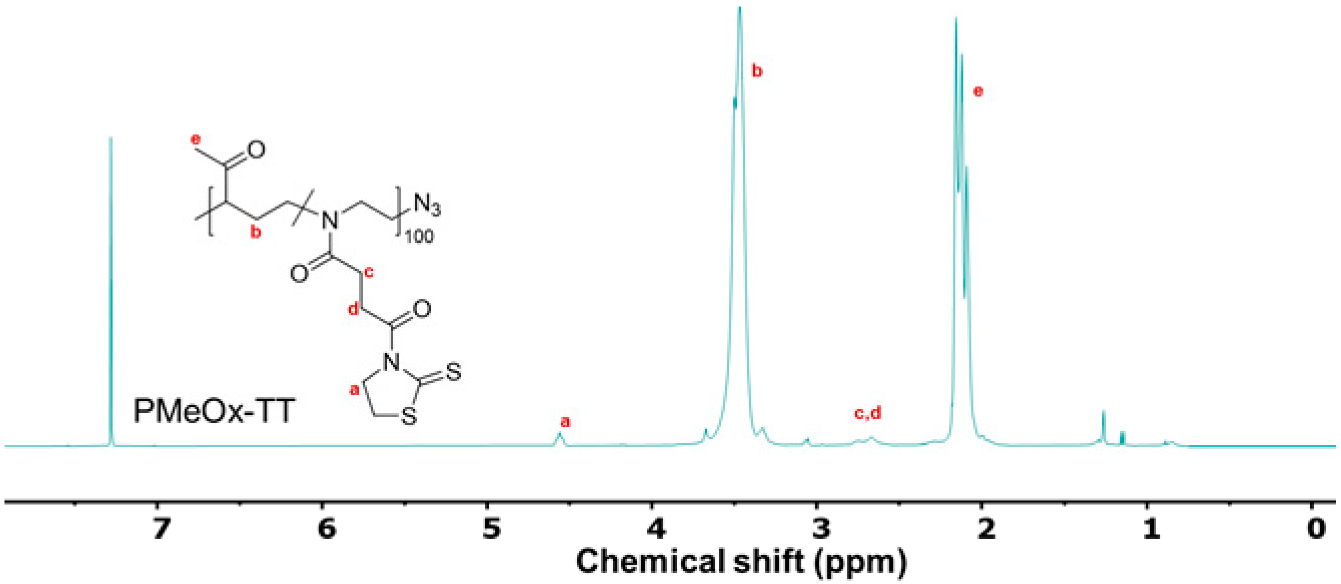

Figure S1. 1H NMR spectra of PMeOx-TT (P8) polymeric precursors. Measured in $CDCl_3$.

Table S1. Results of competitive assay. First, polymers with one terminal hydrophobic anchor, polymers C1, C2 and C3, were coated to the PS surface and BSA with increasing concentration was used to detach the polymer coating.

	C1, 1 mg/L					C2, 1 mg/L					C3, 1 mg/L				
	BSA mg/L														
	0	5	25	50	100	0	5	25	50	100	0	5	25	50	100
	OD_{450}														
	3.028	2.950	1.451	0.948	0.456	2.988	2.615	1.473	1.125	0.706	3.229	2.908	1.696	1.042	0.727
	3.217	2.684	1.623	1.049	0.559	2.795	2.622	1.740	1.128	0.733	3.185	2.796	1.646	1.087	0.812
	3.123	2.588	1.659	1.132	0.634	3.053	2.432	1.830	1.261	0.784	3.054	2.651	1.594	1.116	0.825
	2.965	2.822	1.745	1.193	0.743	2.777	2.791	1.697	1.213	0.827	2.895	2.789	1.734	1.210	0.900
	3.219	2.798	1.796	1.170	0.683	3.005	2.550	1.891	1.424	0.756	3.103	2.712	1.839	1.173	0.897
	3.234	2.757	1.606	1.151	0.728	2.690	2.642	1.731	1.313	0.817	3.248	2.639	1.870	1.167	0.922
	3.039	2.770	1.802	1.092	0.694	2.852	2.677	1.805	1.316	0.718	3.049	2.629	1.983	1.290	0.865
	3.207	2.670	1.782	1.212	0.652	2.852	2.634	1.662	1.120	0.990	3.247	2.819	1.846	1.211	0.679
mean	3.129	2.755	1.683	1.118	0.644	2.876	2.620	1.728	1.237	0.791	3.126	2.739	1.776	1.162	0.828
C.V.,%	*3.37*	*4.00*	*7.25*	*7.76*	*14.81*	*4.41*	*3.91*	*7.37*	*8.97*	*11.58*	*3.99*	*3.62*	*7.34*	*6.77*	*10.49*
Ratio	1.00	0.88	0.54	0.36	0.21	1.00	0.91	0.60	0.43	0.28	1.00	0.88	0.57	0.37	0.26

Table S2. Results of competitive assay. First, polymers with one multiple presentation of hydrophobic anchor, polymers C7, C8 and C9, were coated to the PS surface and BSA with increasing concentration was used to detach the polymer coating.

	**C7, 0.1 mg/L**					**C8, 0.1 mg/L**					**C9, 0.1 mg/L**				
	**BSA mg/L**														
	**0**	**5**	**25**	**50**	**100**	**0**	**5**	**25**	**50**	**100**	**0**	**5**	**25**	**50**	**100**
	**OD_{450}**														
	2.758	2.461	1.431	1.007	0.740	2.486	2.279	1.038	0.843	0.596	2.421	1.145	0.948	0.843	0.809
	2.780	2.304	1.338	1.007	0.709	2.386	2.022	0.978	0.833	0.593	2.227	1.467	0.835	0.816	0.758
	2.733	2.365	1.371	1.030	0.744	2.486	2.047	0.932	0.761	0.580	2.233	1.424	0.869	0.832	0.788
	2.635	2.339	1.495	1.053	0.768	2.433	2.252	1.131	0.782	0.648	2.383	1.517	0.961	0.901	0.785
	2.755	2.366	1.463	1.066	0.762	2.578	1.984	0.979	0.847	0.574	2.284	1.493	0.945	0.895	0.841
	2.743	2.412	1.462	1.018	0.780	2.650	2.151	1.108	0.809	0.618	2.392	1.475	0.947	0.864	0.778
	2.852	2.450	1.482	1.071	0.837	2.664	1.953	1.163	0.746	0.617	2.284	1.562	0.656	0.882	0.760
	2.835	2.388	1.479	1.061	0.842	2.638	2.197	1.070	0.585	0.641	2.323	1.554	0.922	0.838	0.825
**mean**	**2.761**	**2.386**	**1.440**	**1.039**	**0.773**	**2.540**	**2.110**	**1.050**	**0.776**	**0.608**	**2.318**	**1.454**	**0.885**	**0.859**	**0.793**
C.V.,%	***2.41***	***2.25***	***3.94***	***2.56***	***6.02***	***4.20***	***5.95***	***7.83***	***11.09***	***4.51***	***3.19***	***9.16***	***11.60***	***3.65***	***3.76***
**Ratio**	**1.00**	**0.86**	**0.50**	**0.38**	**0.28**	**1.00**	**0.83**	**0.46**	**0.31**	**0.24**	**1.00**	**0.63**	**0.38**	**0.37**	**0.34**
